# Responsiveness of the Japanese Osteoporosis Quality of Life questionnaire in women with postmenopausal osteoporosis

**DOI:** 10.1186/s12955-014-0178-0

**Published:** 2014-12-12

**Authors:** Hisashi Urushihara, Kousei Yoh, Etsuro Hamaya, Masanori Taketsuna, Kiyoshi Tanaka

**Affiliations:** Division of Drug Development & Regulatory Science, Faculty of Pharmacy, Keio University, Minato-ku, Tokyo Japan; Faculty of Health Science, Aino University, Osaka, Japan; Medical Science, Medicines Development Unit Japan, Eli Lilly Japan K.K., Kobe, Japan; Asia Pacific Statistical Sciences, Medicines Development Unit Japan, Eli Lilly Japan K.K., Sannomiya Plaza Building, 7-1-5 Isogamidori, Chuo-ku, Kobe, 651-0086 Japan; Department of Food and Nutrition, Faculty of Home Economics, Kyoto Women’s University, Kyoto, Japan

**Keywords:** JOQOL, Osteoporosis, Quality of Life, Raloxifene, Validation

## Abstract

**Background:**

The Japanese Osteoporosis Quality of Life (JOQOL) questionnaire measures quality of life in Japanese patients with osteoporosis. However, several important aspects of the psychometric properties of individual domains, including responsiveness, have not been addressed to enable valid clinical application. This analysis examined the internal and external responsiveness of the JOQOL questionnaire.

**Methods:**

This was a post hoc analysis of a 24-week prospective postmarketing study of raloxifene (60 mg/day) administered to postmenopausal Japanese women with osteoporosis (JapicCTI-070465). Internal responsiveness was assessed using Standardized Response Mean (SRM) statistics and changes in JOQOL domain scores. Patients were also stratified into those who did or did not achieve a minimal clinically important change (MCIC) in pain, assessed by a visual analogue scale for pain (VAS pain): comparisons were made between treated patients who achieved VAS pain reduction ≥ 20 mm versus VAS pain reduction < 20 mm. External responsiveness was assessed using Pearson’s correlation coefficient (*r*) for changes in JOQOL domain scores with Short Form-8 Health Survey and European Quality of Life Instrument scores.

**Results:**

Of 506 patients analyzed, 421 had a baseline value for VAS pain; of these, 152 patients (36.1%) had a MCIC, whereas 264 patients (62.7%) did not. The JOQOL domains pain, overall health, and falls/psychological factors had small to moderate SRM values (0.3-0.5) in all patients, but consistently showed significantly larger changes in patients whose pain score changes exceeded the MCIC. Together, these findings suggest some degree of internal responsiveness for these domains. However, activities of daily living domain had a SRM value as low as 0.2, and recreation/social activities and posture/physique domains had SRM values close to 0. Moderate correlation (defined as *r* ≥ 0.4 to < 0.6) was noted between the domains pain, activities of daily living, and overall health and some Short Form-8 Health Survey subscales and the European Quality of Life total score, suggesting external responsiveness of these domains.

**Conclusions:**

The inconsistent responsiveness among individual JOQOL domains in treated patients suggests the need for improving several JOQOL domains, especially the activities of daily living, recreation/social activities and posture/physique domains, before application to clinical research.

**Electronic supplementary material:**

The online version of this article (doi:10.1186/s12955-014-0178-0) contains supplementary material, which is available to authorized users.

## Background

Osteoporosis negatively affects quality of life (QOL) as well as clinical and laboratory indices, such as fractures and bone mineral density [1]. Improvement in QOL is therefore an important endpoint for assessing the effectiveness of osteoporosis treatment from patients’ perspectives. Reduced QOL in patients with osteoporosis is primarily caused by fractures, particularly of the spine or hip, which cause pain and impair physical function, social function, and well-being [2–4].

The Japanese Osteoporosis Quality of Life (JOQOL) questionnaire was developed to measure QOL in Japanese patients with osteoporosis, with reference to two English language disease-specific QOL questionnaires: the Qualeffo-41 questionnaire and the Osteoporosis Assessment Questionnaire [3,5–7]. The current JOQOL evaluates the health-related QOL of Japanese patients with osteoporosis using 38 question items grouped into six domains (pain, activities of daily living, recreation/social activities, overall health, posture/physique, and falls/psychological factors). Since its initial development, the JOQOL has been used in a relatively small number of clinical studies [8–10]. The use of the JOQOL in these studies is based on an early validation study that reported high reliability and moderate correlation of the JOQOL total score with the Medical Outcomes Study 36-Item Short-Form Health Survey (SF-36) [7].

Patient-reported outcome (PRO) measures can provide evidence of a treatment benefit from patients’ perspectives [11]. However, for such data to be meaningful, it is necessary that the PRO instrument effectively measures the concept under investigation. Further, before application to clinical trials evaluating treatment effects, the PRO must fulfill appropriate psychometric properties, such as reliability, validity, the ability to detect change in a measure (responsiveness), and interpretability (eg, minimal clinically important change). Construct validity is the ability of an instrument to accurately measure the construct it was designed for [11,12], defined as how ‘a new instrument relates to other tests or measures in the way one would expect if it is really measuring what it is supposed to measure’ [13]. Responsiveness is one important aspect of construct validity, especially for estimating the true treatment effect. Responsiveness is determined by assessing the relationship between changes in established endpoints and those of the PRO measure over time [14].

Kumamoto et al. investigated part of the psychometric properties of the overall JOQOL (total score), including several aspects of reliability and validity [15]. However, they did not examine the validity or reliability of the individual JOQOL domains. In addition, information about other psychometric properties, including responsiveness, was lacking. Because the JOQOL comprises multiple, discrete constructs, further validation of the JOQOL, especially by domain, is warranted before its practical application to clinical studies.

We previously reported significant improvement in impaired QOL in postmenopausal women receiving raloxifene using two established generic QOL instruments: the Short Form-8 Health Survey (SF-8), and the European Quality of Life Instrument (EQ-5D), as well as a visual analogue scale for pain (VAS pain) and the JOQOL questionnaire in a postmarketing study [16]. The aim of the present study was to examine the psychometric properties of the JOQOL questionnaire in this raloxifene-treated population with specific focus on individual domains and responsiveness using the same dataset.

## Methods

### Study design

This post hoc analysis examined the psychometric properties of the JOQOL using data from a previous postmarketing study. The 24-week prospective postmarketing study of raloxifene administered in a real-world clinical practice setting was conducted between 1 September 2007 and 28 February 2009 at 60 hospitals in Japan (Japan Pharmaceutical Information Center registration number JapicCTI-070465) [16].

The postmarketing study population and inclusion/exclusion criteria have been reported previously [16]. The 24-week observation period for the postmarketing study was based on a preceding prospective observational study in postmenopausal women, in which the greatest decrease in diffuse pain (considered to be caused by high bone turnover [17]) was observed at 24 weeks [18]. Further, in the postmarketing study, statistically significant improvements were observed in all QOL instruments (SF8, EQ-5D, and VAS pain) after 8 and 24 weeks of raloxifene treatment [16]. Therefore, we considered it appropriate to conduct this post hoc analysis using the 24-week data of the postmarketing study. The primary objective of the postmarketing study was to assess QOL during the administration of raloxifene (60 mg daily; Evista®, Eli Lilly and Company, Indianapolis, USA) using the SF-8, the EQ-5D, two pain scales including VAS-pain, and the JOQOL. The SF-8 was used for the power calculation and chosen as the primary endpoint for the postmarketing study as it is known to be equivalent to the SF-36 [19] while reducing the burden for patients as it can be completed in one to two minutes.

The postmarketing study was approved by the Institutional Review Board of each participating hospital and conducted in accordance with the Declaration of Helsinki and all applicable local laws and regulations. Informed consent was obtained from the study patients.

### Outcome measures

#### Pain

Pain is the major symptomatic complaint in patients with osteoporosis [2]. Pain intensity is a quantitative estimate of the severity of perceived pain and is most commonly assessed using VAS pain [20]. Therefore, in osteoporosis research, VAS pain can represent a subjective measure for patients’ global assessment of changes in health status in relation to the treatment effects of osteoporosis drugs. In fact, pain intensity measured by a VAS was shown to be significantly alleviated during raloxifene treatment in the postmarketing study [16]. Before starting the postmarketing study, a VAS pain reduction ≥ 20 mm was chosen preliminarily to represent a minimal clinically important change (MCIC) from the patients’ perspective [20]. Patients were stratified into two groups: (i) patients achieving the MCIC (VAS pain reduction ≥ 20 mm) and (ii) patients not achieving the MCIC (VAS pain reduction < 20 mm).

#### Responsiveness

Responsiveness was assessed using two different approaches. Internal responsiveness is the ability of a PRO measure to change over the observation period when treatment-related changes in health status are ‘established’ and is assessed using a distribution-based index such as effect size [14,21]. Our previous report of this study showed that significant improvement was seen in back pain, joint pain, and diffuse bone pain after 8 weeks of raloxifene treatment in the entire treatment group and was greatest at 24 weeks. The other generic QOL instruments, including SF-8 and EQ-5D, showed significant improvements from the baseline values at the 8 weeks of raloxifene treatment and were again greatest at the 24 weeks [16]. Therefore, in the present analysis we used (i) Standardized Response Mean (SRM) values to assess internal responsiveness [21] of the individual JOQOL domains and (ii) changes in JOQOL domain scores over 24 weeks (last observation carried forward; LOCF). Changes in patients were compared between subgroups stratified according to the MCIC [22]. This was done to see whether each domain of the QOL instruments would be responsive in patients with a VAS pain reduction ≥ 20 mm but not in patients with a VAS pain reduction < 20 mm during osteoporosis treatment [16]. External responsiveness is the extent to which changes in a measure under investigation correlate with changes in a validated (‘external’) reference measure, such as an established QOL instrument [21]. External responsiveness is assessed by anchor-based methods that use appropriate external criteria as ‘anchors’ [14]. These external criteria may be clinical endpoints, patient-rated global improvement measures, or other PROs with established responsiveness. A meaningful change in an established PRO measure that correlates with a change in the PRO measure under investigation suggests that the change in the investigation measure is also meaningful [23]. External responsiveness of JOQOL domains were verified against two external anchors: the SF-8 (subscale and summary scores) and the EQ-5D (total score) via correlation analysis. As statistically significant changes were observed in all SF-8 domain scores and EQ-5D score for the whole group in the postmarketing study [16], the SF-8 can be considered a responsive measurement in this population.

### Statistical analyses

Summary statistics for patient characteristics and the JOQOL scores were determined as mean ± standard deviation (SD). For internal responsiveness assessment, changes in JOQOL domain scores over the observation period were compared between patients stratified by MCIC and assessed using the Student t-test with a two-sided alpha level of 0.05. The SRM is the mean of the difference of the change in JOQOL domain scores divided by the SD of the difference; SRM values of ≥ 0.2 to < 0.5, ≥ 0.5 to < 0.8, and ≥ 0.8 represented small, moderate, and large internal responsiveness, respectively [21]. For external responsiveness, Pearson’s correlation coefficients (*r*) were calculated for changes in JOQOL domains and changes in the external anchors [21]. Correlation coefficients of < 0.4, ≥ 0.4 to < 0.6, and ≥ 0.6 to < 0.8 represented weak, moderate, and strong correlation, respectively [24]. Statistical analyses were conducted using SAS Version 9.1.3 (SAS Institute, Cary, NC, USA).

## Results

### Patient disposition and baseline characteristics

Of the 536 patients enrolled in the postmarketing study, 506 were eligible for analysis (30 patients excluded; 26 patients did not complete at least one follow-up visit, 1 patient violated concomitant drug protocol, 3 patients had no case report form) [16]. In brief, patients were postmenopausal women aged between 45 and 99 years. The mean time since menopause was 21.4 years and the mean period of treatment was 163.5 days (Table 1).

**Table 1:** **Patient characteristics**

**Characteristic**	***n***	**Mean (SD)**
Age, years	506	70.7 (8.7)
Height, cm	419	150.2 (6.6)
Maximum height at adulthood, cm	156	153.1 (5.4)
Weight, kg	417	50.8 (8.4)
Body mass index, kg/m^2^	411	22.6 (3.5)
Years since menopause, years	217	21.4 (9.5)
Treatment period, days	506	163.5 (53.3)

Abbreviation: *SD* standard deviation.

### Pain

For the entire treatment group, VAS pain was reduced by 11.7 ± 25.8 mm (n = 416) from a baseline value of 40.2 ± 24.9 mm (n = 421), which corresponded to a moderate SRM value of 0.45. Stratification of patients according to the MCIC criteria showed that 152/421 patients (36.1%) had a VAS pain reduction ≥ 20 mm (ie, achieved the MCIC). These patients had a baseline mean ± SD VAS pain score of 58.4 ± 16.8 mm, a mean ± SD reduction in VAS pain of 38.7 ± 16.2 mm. In contrast, 264/421 patients (62.7%) had a VAS pain reduction < 20 mm (ie, did not achieve the MCIC). These patients had a mean ± SD baseline VAS pain score of 30.0 ± 22.5 mm and a mean ± SD change in VAS pain of -3.8 ± 15.3 mm, indicating no reduction in pain status and a significant difference with those who achieved the MCIC (*p* < 0.001). These findings confirmed that the predefined MCIC criteria differentiated clearly between Japanese patients with osteoporosis who experienced clinically significant changes in health status related to perceived pain and those without such perceived changes in pain.

### Internal responsiveness of the JOQOL

The mean evaluation period for the different domains of the JOQOL was consistent and ranged between 172.3 ± 11.4 days (range 117 to 196 days) and 174.3 ± 12.0 days (range 120 to 196 days). For the entire treatment group, the JOQOL domains including pain, activities of daily living, overall health, and falls/psychological factors and the total score showed small responsiveness with regard to SRM values (0.4, 0.2, 0.5, 0.3, and 0.4, respectively; Table 2). However, no responsiveness was observed for the domains of recreation/social activities and posture/physique for the entire treatment group (0.0 and 0.1, respectively). After the stratification, the JOQOL domains pain, activities of daily living, overall health, and falls/psychological factors, and the total score had significantly larger changes for patients achieving the MCIC compared with patients not achieving the MCIC (*p* < 0.001; Table 3). Taken together, the above results show some degree of internal responsiveness for the domains pain, overall health, falls/psychological factors, and the total score, however, the SRM for activities of daily living indicated low internal responsiveness. For two JOQOL domains (recreation/social activities and posture/physique; Table 3), the SRM values were consistently < 0.2 and the differences in mean changes in scores between groups stratified by the MCIC criteria for these domains were not statistically significant. For the SF-8 subscales and the EQ-5D, small but consistent responsiveness (SRM values 0.3-0.4) were observed in the entire treatment group (Additional file 1). In patients achieving the MCIC, significantly larger changes was noted for all the SF-8 subscales (*p* ≤ 0.001 for all subscales) and the EQ-5D total score (*p* < 0.001) (Additional file 2).

**Table 2:** **Baseline and change in scores and internal responsiveness of the JOQOL in postmenopausal Japanese women with osteoporosis for the entire treatment group**

**Domain**	**Baseline**		Change in score and internal responsiveness at 24 weeks*		
	**n**	**Mean (SD)**	**n**	**Mean (SD)**	**SRM**
Pain	407	14.1 (4.6)	277	1.9 (4.4)	0.4
Activities of daily living	416	51.2 (12.8)	280	1.6 (8.7)	0.2
Recreation/social activities	404	9.0 (4.8)	271	0.0 (4.2)	0.0
Overall health	436	5.1 (2.1)	309	1.0 (2.2)	0.5
Posture/physique	405	10.5 (3.8)	267	0.3 (2.7)	0.1
Falls/psychological factors	423	12.0 (4.1)	300	0.9 (3.3)	0.3
Total score	343	66.9 (15.8)	216	4.0 (11.5)	0.4

Abbreviations: *JOQOL* Japanese Osteoporosis Quality of Life questionnaire, *SD* standard deviation, *SRM* standardized response mean.

*Change from baseline to last observation carried forward.

**Table 3:** **Baseline and change in scores of the JOQOL in postmenopausal Japanese women with osteoporosis stratified by minimal clinically important change in VAS pain**

	**MCIC (VAS pain reduction)**	**Baseline**		Change in score at 24 weeks*		
		***n***	**Mean (SD)**	***n***	**Mean (SD)**	***p*** **value** ^**†**^
Pain	≥ 20 mm	145	12.3 (4.4)	109	4.1 (4.7)	< 0.001
	< 20 mm	248	14.9 (4.4)	163	0.3 (3.5)	
Activities of daily living	≥ 20 mm	148	46.0 (13.4)	110	6.1 (9.3)	< 0.001
	< 20 mm	250	53.8 (11.8)	165	-1.3 (7.1)	
Recreation/social activities	≥ 20 mm	143	8.2 (4.8)	104	0.4 (4.3)	0.215
	< 20 mm	242	9.3 (4.8)	160	-0.2 (4.1)	
Overall health	≥ 20 mm	152	4.7 (2.0)	118	1.7 (2.4)	< 0.001
	< 20 mm	264	5.4 (2.2)	183	0.6 (2.1)	
Posture/physique	≥ 20 mm	146	9.8 (3.6)	109	0.6 (2.7)	0.219
	< 20 mm	241	10.8 (3.9)	153	0.2 (2.7)	
Falls/psychological factors	≥ 20 mm	151	11.3 (4.1)	116	1.8 (3.6)	< 0.001
	< 20 mm	254	12.4 (4.0)	177	0.2 (3.0)	
Total score	≥ 20 mm	133	61.0 (15.7)	93	9.7 (12.2)	< 0.001
	< 20 mm	201	70.4 (14.8)	120	-0.3 (9.0)	

Abbreviations: *JOQOL* Japanese Osteoporosis Quality of Life questionnaire, *MCIC* minimal clinically important change, *SD* standard deviation, *SRM* standardized response mean, *VAS* visual analogue scale.

*Change from baseline to last observation carried forward; ^**†**^Determined by two-sample t-test**.**

### External responsiveness of the JOQOL

Moderate correlation was noted for changes in the JOQOL domains pain, activities of daily living, overall health, and the total score with changes in the external anchors (ie, the various SF-8 subscales and the EQ-5D total score; Table 4). Specifically, there was moderate correlation for the JOQOL domain activities of daily living and the SF-8 subscale general health (*r* = 0.43), the JOQOL domain overall health and the SF-8 subscale general health (*r* = 0.43), and the JOQOL domain pain and the SF-8 subscale role physical (*r* = 0.40). Moderate correlation was also noted for the changes in the JOQOL activities of daily living and changes in the SF-8 physical component summary score (*r* = 0.43) and the EQ-5D total score (*r* = 0.52). Overall, weak correlation was noted for changes in the JOQOL domains recreation/social activities, posture/physique, and falls/psychological factors with changes in any of the SF-8 subscales or the EQ-5D total score (*r* < 0.4 for all correlations). Of note, the correlation values of each individual JOQOL domain with the changes in various SF-8 subscales were consistent. This is contrary to the expectation that a domain-specific correlation pattern that reflected the discrete SF-8 constructs and the construct of each JOQOL domain would be observed.

**Table 4:** **Correlation of changes in JOQOL scores with changes in external QoL measures (SF-8 and EQ-5D)** ^**a**^

**JOQOL**	**SF-8**										**EQ-5D**
**Domain**	**GH**	**PF**	**RP**	**BP**	**VT**	**SF**	**MH**	**RE**	**PCS**	**MCS**	
Pain	0.35	0.32	0.40	0.36	0.34	0.33	0.38	0.35	0.38	0.33	0.39
Activities of daily living	0.43	0.38	0.41	0.38	0.34	0.42	0.35	0.42	0.43	0.34	0.52
Recreation/social activities	0.12	0.13	0.18	0.05	0.15	0.26	0.20	0.28	0.08	0.27	0.11
Overall health	0.43	0.22	0.30	0.32	0.39	0.27	0.31	0.27	0.32	0.30	0.36
Posture/physique	0.09	0.07	0.15	0.20	0.13	0.20	0.22	0.18	0.11	0.21	0.05
Falls/psychological factors	0.29	0.29	0.35	0.32	0.25	0.31	0.35	0.29	0.32	0.28	0.30
Total score	0.50	0.44	0.50	0.46	0.42	0.53	0.55	0.59	0.48	0.52	0.51

^a^In postmenopausal women with osteoporosis (*n* = 212 to 299).

Abbreviations: *BP* bodily pain, *EQ-5D* European Quality of Life Instrument, *GH* general health, *JOQOL* Japanese Osteoporosis Quality of Life questionnaire, *MCS* mental component summary, *MH* mental health, *PCS* physical component summary, *PF* physical functioning, *QoL* quality of life, *RE* role emotional, *RP* role physical, *SF* social functioning, *SF-8* Short Form-8 Health Survey, *VT* vitality.

## Discussion

To our knowledge, this is the first study to examine the responsiveness of the JOQOL, a disease-specific QOL questionnaire for Japanese women with osteoporosis. Some of the JOQOL domains tested in the present analysis including pain, fall/psychological factors, and overall health showed limited internal responsiveness, comparable to the established SF-8 subscales and EQ-5D. However, the activities of daily living, recreation/social activities and posture/physique domains were found to be less- or non-responsive in regard to internal responsiveness. These findings for JOQOL domain internal responsiveness were largely consistent with the results of the external responsiveness analysis. Our results indicate that there may be further room for scrutinizing and improving the JOQOL, a disease-specific QOL instrument developed to measure the treatment effectiveness of osteoporosis medications.

Certain JOQOL domain scores (pain, activities of daily living, overall health, falls/psychological factors) and the total score showed small internal responsiveness among the treatment population. These were fairly consistent with the significantly larger changes among patients with a VAS pain reduction ≥ 20 mm. In contrast, the JOQOL domains recreation/social activities and posture/physique failed to show adequate internal responsiveness with regard to the SRM values and between-group differences.

Assessment of external responsiveness also found moderate correlation between the JOQOL domains pain, activities of daily living, overall health, and the total score and various subscales of the SF-8 and the EQ-5D total score as references. However, homogeneous, non-specific correlation patterns for changes in individual JOQOL domains were observed regardless of the constructs measured by the SF-8 subscales. This non-specific correlation pattern did not support the expectation that a domain-specific correlation pattern between the SF-8 constructs and the JOQOL domains would be observed [7].

The findings of this study partly support the validity of several JOQOL domains in terms of responsiveness for detecting changes over time in QOL and subjective symptoms in patients with osteoporosis. However, the finding that the JOQOL domains recreation/social activities and posture/physique failed to show sufficient internal or external responsiveness requires further investigation. Additionally, the internal responsiveness index for activities of daily living had an SRM value as low as 0.2. Insufficient responsiveness may be because these domains were designed to measure concepts that are not specifically related to subjective pain, although pain was previously shown to change during the course of raloxifene treatment and was assumed to represent an improvement in patient global assessment [16]. Indeed, the JOQOL domains recreation/social activities and posture/physique showed very low correlation with the SF-8 bodily pain subscale, suggesting these particular JOQOL domains are unrelated to pain. An earlier study conducted in Japan that investigated the change in QOL in osteoporosis patients with back pain over six months supports this finding [25]. In the postmarketing study, however, raloxifene treatment was associated with improvements in scores for all SF-8 subscales and the EQ-5D [16]. Therefore, the treatment effects observed in the postmarketing study were considered to affect all aspects of health-related constructs covered by the generic QOL measures (SF-8 and EQ-5D), rather than just pain [19,26,27]. Alternatively, it is possible that a ceiling effect (skewed distribution around the upper limit of the scale) may be responsible, [12] at least in part, for the lack of responsiveness observed for the JOQOL domains recreation/social activities and posture/physique. However, this alternative explanation seems unlikely because, for both domains, middle-range scores were present at baseline, which suggests that there was potential for a change in scores.

The internal responsiveness analysis found that pain relief was not associated with improvement in the JOQOL recreation/social activities domain. This is contrary to the fact that the SF-8 social functioning subscale, which is expected to have a similar construct to the JOQOL recreation/social activities, responded significantly in treated patients [16]. Further, the JOQOL recreation/social activities domain showed low correlation with the SF-8 social functioning subscale. These findings suggest that the JOQOL recreation/social activities domain may have a different construct from the ‘established’ SF-8 social functioning construct as well as questionable construct validity in terms of responsiveness as an osteoporosis-specific measure.

Similarly, the JOQOL domain posture/physique also appears to have inadequate construct validity as an osteoporosis-specific measure based on its unresponsiveness to treatment. The JOQOL domain posture/physique comprises items related to supposedly incurable symptoms, including shortening of height and hunched back. Further, the lack of correlation of the posture/physique domain with any of the SF-8 subscales/summary score and EQ-5D total score suggests that this domain was not sufficiently sensitive to any treatment-related changes in health-related QOL from the patients’ perspectives.

A low SRM value for activities of daily living, representing weak internal responsiveness, appears to be ascribed to a larger SD of the change score. On the contrary, the between-group difference of this domain stratified by the MCIC criteria was large and significant, and moderate or better correlations with external anchors such as the SF-8 general health, social functioning, role emotional and role physical subscales, and the EQ-5D score were noted in the external responsiveness analysis compared with other JOQOL domains. This inconsistency warrants further investigation.

The conceptual framework of the JOQOL suggests that the individual JOQOL domains should have correlated in a domain-specific manner with the individual SF-8 subscales [7]. That is, a domain-specific correlation pattern for certain JOQOL domains and individual SF-8 subscales that measure the same construct would be expected so that SF-8 subscales with similar concepts should be correlated (convergent validity), but SF-8 subscales with divergent concepts should not be correlated (discriminant validity) [12]. The weak specificity of correlations observed implies suboptimal construct validity of the JOQOL domains and the possible need to re-examine the conceptual framework of the JOQOL. Further improvement in the construct validity of each JOQOL domain by reliability and/or factor analysis appears warranted before formal clinical application of this instrument.

There are several notable strengths and limitations of the present study. In terms of study strengths, the results of internal and external responsiveness were consistent, which confers robustness on the results. Further, we used a sample population that was sufficiently large to determine responsiveness to treatment effects with osteoporosis medications in a real-world setting. Although this confers generalizability to postmenopausal women with osteoporosis, our results can only apply to postmenopausal women with osteoporosis in Japan. This is because treatment responsiveness and MCIC can only be generalized to the population studied [14]. Hence, a separate validation study looking at men with osteoporosis in Japan is warranted in this regard. Finally, we relied primarily on the pain-related changes to establish “subjective global changes” during raloxifene treatment in the assessment of responsiveness as discussed above. Therefore, the other aspects of QOL unrelated to pain may be insufficiently considered in the assessment of the responsiveness of the JOQOL domains.

## Conclusions

This is the first report of the validity of individual domains of the JOQOL questionnaire among Japanese women with osteoporosis for the purpose of measuring treatment effects. Pain, overall health, falls/psychological factors, and total score of the JOQOL seem to have limited responsiveness to treatment changes. Activities of daily living, recreation/social activities and posture/physique domains were less responsive based on SRM values than generic QOL measures, such as the SF-8 and EQ-5D. Construct validity of the JOQOL domains also seems to be problematic because all the domains of the JOQOL were correlated with all aspects of health-related QOL covered by SF-8 in an non-specific manner, possibly indicating lack of convergent and discriminant validity. Thus, improvements in the JOQOL are suggested before application to clinical research for osteoporosis. Specifically, scrutiny of the constructs and content (especially those related to activities of daily living, recreation/social activities and posture/physique), and factor analysis of the question items comprising these domains may be recommended.

## Additional files

Additional file 1:
**Baseline and change in SF-8 and EQ-5D in postmenopausal women with osteoporosis for the entire treatment group.**
Additional file 2:
**Baseline and change in scores of the SF-8 and EQ-5D in postmenopausal Japanese women with osteoporosis stratified by minimal clinically important change in VAS pain.**

## Abbreviations

EQ-5D: European Quality of Life Instrument
JOQOL: Japanese Osteoporosis Quality of Life (questionnaire)
LOCF: last observation carried forward
MCIC: Minimal Clinically Important Change
MCS: Mental Component Summary
PCS: Physical Component Summary
PRO: Patient-Reported Outcome
QOL: Quality of Life
SD: Standard Deviation
SF-8: Short Form-8 Health Survey
SF-36: Medical Outcomes Study 36-Item Short-Form Health Survey
SRM: Standardized Response Mean
VAS: Visual Analogue Scale (for pain)

## References

[1] Orimo H, Nakamura T, Hosoi T, Iki M, Uenishi K, Endo N, Ohta H, Shiraki M, Sugimoto T, Suzuki T, Soen S, Nishizawa Y, Hagino H, Fukunaga M, Fujiwara S (2012). Japanese 2011 guidelines for prevention and treatment of osteoporosis–executive summary. Arch Osteoporos.

[2] Adachi JD, Adami S, Gehlbach S, Anderson FA, Boonen S, Chapurlat RD, Compston JE, Cooper C, Delmas P, Diez-Perez A, Greenspan SL, Hooven FH, LaCroix AZ, Lindsay R, Netelenbos JC, Wu O, Pfeilschifter J, Roux C, Saag KG, Sambrook PN, Silverman S, Siris ES, Nika G, Watts NB, Glow Investigators (2010). Impact of prevalent fractures on quality of life: baseline results from the global longitudinal study of osteoporosis in women. Mayo Clin Proc.

[3] Lips P, van Schoor NM (2005). Quality of life in patients with osteoporosis. Osteoporos Int.

[4] Tanaka K, Yoshizawa M, Yoh K (2005). Improvement of QOL in osteoporotic patients by calcitonin treatment (in Japanese). Clin Calcium.

[5] Lips P, Cooper C, Agnusdei D, Caulin F, Egger P, Johnell O, Kanis JA, Liberman U, Minne H, Reeve J, Reginster JY, de Vernejoul MC, Wiklund I (1997). Quality of life as outcome in the treatment of osteoporosis: the development of a questionnaire for quality of life by the European Foundation for Osteoporosis. Osteoporos Int.

[6] Silverman SL (2000). The Osteoporosis Assessment Questionnaire (OPAQ): a reliable and valid disease-targeted measure of health-related quality of life (HRQOL) in osteoporosis. Qual Life Res.

[7] Takahashi H, Iwaya T, Iba K, Gorai I, Suzuki T, Hayashi Y, Fujinawa O, Yamazaki K, Endo N (2001). A trial of the Japanese Osteoporosis Quality of Life questionnaire 1999 version and a development of 2000 version (in Japanese). Jpn J Bone Metab.

[8] Kawate H, Ohnaka K, Adachi M, Kono S, Ikematsu H, Matsuo H, Higuchi K, Takayama T, Takayanagi R (2010). Alendronate improves QOL of postmenopausal women with osteoporosis. Clin Interv Aging.

[9] Miyakoshi N, Itoi E, Kobayashi M, Kodama H (2003). Impact of postural deformities and spinal mobility on quality of life in postmenopausal osteoporosis. Osteoporos Int.

[10] Shiraki M, Kuroda T, Miyakawa N, Fujinawa N, Tanzawa K, Ishizuka A, Tanaka S, Tanaka Y, Hosoi T, Itoi E, Morimoto S, Itabashi A, Sugimoto T, Yamashita T, Gorai I, Mori S, Kishimoto H, Mizunuma H, Endo N, Nishizawa Y, Takaoka K, Ohashi Y, Ohta H, Fukunaga M, Nakamura T, Orimo H (2011). Design of a pragmatic approach to evaluate the effectiveness of concurrent treatment for the prevention of osteoporotic fractures: rationale, aims and organization of a Japanese Osteoporosis Intervention Trial (JOINT) initiated by the Research Group of Adequate Treatment of Osteoporosis (A-TOP). J Bone Miner Metab.

[11] U. S. Department of Health Human Services (2006). Guidance for industry: patient-reported outcome measures: use in medical product development to support labeling claims: draft guidance. Health Qual Life Outcomes.

[12] Fayers P, Machin D (2000). Quality of life: assessment, analysis, and interpretation.

[13] Guyatt G, Walter S, Norman G (1987). Measuring change over time: assessing the usefulness of evaluative instruments. J Chronic Dis.

[14] Revicki DA, Cella D, Hays RD, Sloan JA, Lenderking WR, Aaronson NK (2006). Responsiveness and minimal important differences for patient reported outcomes. Health Qual Life Outcomes.

[15] Kumamoto K, Nakamura T, Suzuki T, Gorai I, Fujinawa O, Ohta H, Shiraki M, Yoh K, Fujiwara S, Endo N, Matsumoto T (2010). Validation of the Japanese osteoporosis quality of life questionnaire. J Bone Miner Metab.

[16] Yoh K, Hamaya E, Urushihara H, Iikuni N, Yamamoto T, Taketsuna M (2012). Quality of life in raloxifene-treated Japanese women with postmenopausal osteoporosis: a prospective, postmarketing observational study. Curr Med Res Opin.

[17] Treede RD (1999). The physiology of bone pain. Osteologie.

[18] Scharla S, Oertel H, Helsberg K, Kessler F, Langer F, Nickelsen T (2006). Skeletal pain in postmenopausal women with osteoporosis: prevalence and course during raloxifene treatment in a prospective observational study of 6 months duration. Curr Med Res Opin.

[19] Fukuhara S, Suzukano Y (2004). Manual of the SF-8 Japanese version (in Japanese).

[20] Ostelo RW, de Vet HC (2005). Clinically important outcomes in low back pain. Best Pract Res Clin Rheumatol.

[21] Husted JA, Cook RJ, Farewell VT, Gladman DD (2000). Methods for assessing responsiveness: a critical review and recommendations. J Clin Epidemiol.

[22] Juniper EF, Guyatt GH, Ferrie PJ, Griffith LE (1993). Measuring quality of life in asthma. Am Rev Respir Dis.

[23] Urushihara H, Fukuhara S, Tai S, Morita S, Chihara K (2007). Heterogeneity in responsiveness of perceived quality of life to body composition changes between adult- and childhood-onset Japanese hypopituitary adults with GH deficiency during GH replacement. Eur J Endocrinol.

[24] Swinscow TDV: **Statistics at square one: 11. Correlation and regression.** *BMJ* 1997: [http://www.bmj.com/about-bmj/resources-readers/publications/statistics-square-one/11-correlation-and-regression]

[25] Takada J, Iba K, Yamashita T, Katahira G (2004). QOL in osteoporotic patients with vertebral fractures (in Japanese). Clin Calcium.

[26] EuroQol G (1990). EuroQol–a new facility for the measurement of health-related quality of life. Health Policy.

[27] Japanese EuroQol translation team (1998). The development of the Japanese EuroQol instrument. Shakaitorinsho.

