# Supplementary material for: Responsiveness of the Japanese Osteoporosis Quality of Life questionnaire in women with postmenopausal osteoporosis
Source: Health Qual Life Outcomes. 2014 Dec 12;12:178. doi: 10.1186/s12955-014-0178-0 (PMC4279675; doi:10.1186/s12955-014-0178-0)
Supplement: Additional file 1: — Baseline and change in SF-8 and EQ-5D in postmenopausal women with osteoporosis for the entire treatment group. [file 12955_2014_178_MOESM1_ESM.docx]

**Additional file 1 – Baseline and change in SF-8 and EQ-5D in postmenopausal women with osteoporosis for the entire treatment group**

| **Subscale** | **Baseline** | | **Change in score and SRM at 24 weeks*** | | |
| --- | --- | --- | --- | --- | --- |
|  | ***n*** | **Mean (SD)** | ***n*** | **Mean (SD)** | **SRM** |
| *SF-8 Subscales* | | | | | |
| General Health | 434 | 46.0 (7.6) | 432 | 2.8 (8.2) | 0.3 |
| Physical Functioning | 434 | 44.3 (7.3) | 433 | 2.5 (7.5) | 0.3 |
| Role Physical | 432 | 44.1 (8.6) | 431 | 2.9 (8.7) | 0.3 |
| Bodily Pain | 434 | 43.7 (9.1) | 433 | 3.7 (9.1) | 0.4 |
| Vitality | 433 | 48.1 (7.0) | 432 | 2.1 (7.7) | 0.3 |
| Social Functioning | 435 | 44.8 (9.8) | 434 | 2.9 (10.3) | 0.3 |
| Mental Health | 434 | 48.8 (7.2) | 433 | 2.2 (7.4) | 0.3 |
| Role Emotional | 434 | 46.3 (9.0) | 433 | 3.0 (8.5) | 0.4 |
| *SF-8 Component Summary Scores* | | | | | |
| PCS | 428 | 42.0 (7.8) | 426 | 3.2 (7.9) | 0.4 |
| MCS | 428 | 48.5 (7.7) | 426 | 2.0 (7.8) | 0.3 |
| *EQ-5D* | | | | | |
| EQ-5D total score | 428 | 0.7 (0.2) | 427 | 0.1 (0.2) | 0.4 |

Abbreviations: EQ-5D = European Quality of Life Instrument, MCS = mental component summary, PCS = physical component summary, SD = standard deviation, SF-8 = Short Form-8 Health Survey, SRM = standardized response mean.

*Change from baseline to last observation forward.
