# Supplementary material for: Responsiveness of the Japanese Osteoporosis Quality of Life questionnaire in women with postmenopausal osteoporosis
Source: Health Qual Life Outcomes. 2014 Dec 12;12:178. doi: 10.1186/s12955-014-0178-0 (PMC4279675; doi:10.1186/s12955-014-0178-0)
Supplement: Additional file 2: — Baseline and change in scores of the SF-8 and EQ-5D in postmenopausal Japanese women with osteoporosis stratified by minimal clinically important change in VAS pain. [file 12955_2014_178_MOESM2_ESM.docx]

**Additional file 2 – Baseline and change in scores of the SF-8 and EQ-5D in postmenopausal Japanese women with osteoporosis** **stratified by minimal clinically important change in VAS pain**

| **Subscale** | **MCIC (VAS pain reduction)** | **Baseline** | | **Change in score at 24 weeks*** | | |
| --- | --- | --- | --- | --- | --- | --- |
|  |  | ***n*** | **Mean (SD)** | **n** | **Mean (SD)** | ***p* value^†^** |
| *SF-8 Subscales* | | | | | | |
| General Health | ≥ 20 mm | 152 | 43.9 (8.0) | 151 | 5.9 (8.9) | < 0.001 |
|  | < 20 mm | 262 | 47.0 (7.1) | 261 | 1.1 (7.2) |  |
| Physical Functioning | ≥ 20 mm | 151 | 42.3 (7.0) | 151 | 4.8 (7.6) | < 0.001 |
|  | < 20 mm | 263 | 45.2 (7.3) | 262 | 1.2 (7.1) |  |
| Role Physical | ≥ 20 mm | 150 | 40.9 (8.5) | 150 | 6.8 (9.1) | < 0.001 |
|  | < 20 mm | 262 | 45.7 (8.2) | 261 | 0.8 (7.6) |  |
| Bodily Pain | ≥ 20 mm | 152 | 40.4 (8.2) | 152 | 8.4 (9.1) | < 0.001 |
|  | < 20 mm | 262 | 45.3 (8.9) | 261 | 0.9 (8.1) |  |
| Vitality | ≥ 20 mm | 152 | 46.5 (7.2) | 152 | 4.3 (8.0) | < 0.001 |
|  | < 20 mm | 261 | 48.9 (6.6) | 260 | 0.9 (7.2) |  |
| Social Functioning | ≥ 20 mm | 151 | 42.3 (10.2) | 151 | 5.2 (10.8) | 0.001 |
|  | < 20 mm | 263 | 46.2 (9.2) | 262 | 1.6 (10.0) |  |
| Mental Health | ≥ 20 mm | 151 | 47.1 (7.5) | 151 | 4.2 (7.5) | < 0.001 |
|  | < 20 mm | 262 | 49.7 (7.0) | 261 | 1.2 (6.9) |  |
| Role Emotional | ≥ 20 mm | 151 | 44.0 (10.0) | 151 | 5.5 (9.4) | < 0.001 |
|  | < 20 mm | 262 | 47.7 (8.0) | 261 | 1.4 (7.2) |  |
| *SF-8 Component Summary Scores* | | | | | | |
| PCS | ≥ 20 mm | 150 | 39.1 (7.0) | 149 | 7.0 (7.7) | < 0.001 |
|  | < 20 mm | 258 | 43.4 (7.7) | 257 | 1.0 (7.2) |  |
| MCS | ≥ 20 mm | 150 | 47.0 (8.6) | 149 | 3.5 (8.2) | 0.004 |
|  | < 20 mm | 258 | 49.4 (7.1) | 257 | 1.2 (7.5) |  |
| *EQ-5D* | | | | | | |
| EQ-5D total score | ≥ 20 mm | 152 | 0.6 (0.1) | 151 | 0.1 (0.2) | < 0.001 |
|  | < 20 mm | 260 | 0.7 (0.2) | 260 | 0.0 (0.1) |  |

^a^in postmenopausal female Japanese women with osteoporosis.

Abbreviations: EQ-5D = European Quality of Life Instrument, MCIC = minimal clinically important difference, MCS = mental component summary, PCS = physical component summary, SD = standard deviation, SF-8 = Short Form-8 Health Survey, SRM = standardized response mean, VAS = visual analogue scale.

*Change from baseline to last observation carried forward; ^†^Determined by two-sample t-test.
